# Procalcitonin levels among patients with fever secondary to severe intracerebral infection. A cross-sectional study

**DOI:** 10.1590/1516-3180.2018.0458220719

**Published:** 2019-10-31

**Authors:** Sümeyye Selim Kara, Ayhan Akbulut, Ayşe Sağmak Tartar, Hatice Handan Akbulut, Kutbeddin Demirdağ, Azize Beştaş

**Affiliations:** I MD. Attending Physician, Department of Infectious Diseases and Clinical Microbiology, Fırat Üniversitesi Tıp Fakültesi, Elazig, Turkey.; II MD. Professor, Department of Infectious Diseases and Clinical Microbiology, Fırat Üniversitesi Tıp Fakültesi, Elazig, Turkey.; III MD. Associate Professor, Department of Infectious Diseases and Clinical Microbiology, Fırat Üniversitesi Tıp Fakültesi, Elazig, Turkey.; IV MD. Professor, Department of Immunology, Fırat Üniversitesi Tıp Fakültesi, Elazig, Turkey.; V MD. Professor, Department of Infectious Diseases and Clinical Microbiology, Fırat Üniversitesi Tıp Fakültesi, Elazig, Turkey.; VI MD. Professor, Department of Anesthesia and Reanimation, Fırat Üniversitesi Tıp Fakültesi, Elazig, Turkey.

**Keywords:** Fever, Procalcitonin, C-reactive protein

## Abstract

**BACKGROUND:**

Making the differential diagnosis between central fever and infectious fever is critically important among intracerebral hemorrhage patients followed up in intensive care units (ICUs). Serum procalcitonin (PCT) has been found to be a promising biomarker for the initial diagnosis of infection, even before culturing results.

**OBJECTIVES:**

To investigate the relationship between PCT and both fever etiologies and C-reactive protein (CRP) levels among critically ill patients with suspected intracerebral hemorrhage.

**DESIGN AND SETTING:**

Cross-sectional study in a public university hospital in Elazig, Turkey.

**METHODS:**

ICU patients diagnosed with intracerebral hemorrhage and normal procalcitonin levels were included in this study. From clinical assessments and cultures, they were classified as presenting either infectious or central fever. The sensitivity and specificity of PCT and CRP for predicting infection were calculated using a receiver operating characteristic (ROC) curve.

**RESULTS:**

There were 98 ICU patients with diagnoses of intracerebral hemorrhage. The median (interquartile range) PCT levels of patients with infectious and central fever were 4 (0.9-11) and 0.1 (0.1-0.4) ng/ml, respectively, with a statistically significant intergroup difference (P < 0.001). The areas under the ROC curve for predicting infectious or central fever PCT and CRP were 0.958 (P < 0.001) and 0.816 (P < 0.001), respectively. A statistically significant positive correlation was detected between PCT and CRP levels in patients with infectious fever (rho: 0.461; P = 0.003), but not in patients with central fever.

**CONCLUSIONS:**

PCT can possibly be used as a biomarker to differentiate between infectious and central fever among ICU patients.

## INTRODUCTION

In intensive care units (ICUs), delay in diagnosis and treatment increases mortality rates. Serious infections among ICU patients most commonly consist of respiratory tract infections, followed by urinary system infections, wound site infections and primary bacteremia.^1^^,^^2^

Fever in patients hospitalized in ICUs is an important indicator of infection. Despite new treatment alternatives, the infection-related mortality rate remains high. Likewise, clinical findings of fever remain frequent. Moreover, routine laboratory tests are not specific and sometimes may mislead clinicians. Lack of early-phase and specific markers for diagnosing infection cause delays in treatment and unnecessary use of antibiotics.^3^ Early diagnosis and appropriate antibiotherapy decrease infection-related morbidity and mortality.^4^

Assessment of clinical and laboratory findings constitutes an ideal method for diagnosing infection. Growth of an infectious agent through culturing provides the most important laboratory evidence. However, this method is time-consuming. While awaiting the results from culturing, other diagnostic laboratory parameters such as the levels of procalcitonin (PCT), C-reactive protein (CRP), leukocytes, neutrophils and erythrocyte sedimentation rate (ESR) have been used. Over recent years, serum PCT has been found to be an important and promising biomarker for making the initial diagnosis of infection.^5^

The incidence of fever in ICUs is 23%.^6^ Nearly 50% of these cases relate to noninfectious etiologies. The noninfectious causes of fever include bleeding, atelectasis, drug effects, venous thromboembolism and blood transfusion reactions.^7^

Another noninfectious type of fever seen in ICUs is central fever in patients diagnosed with intracerebral hemorrhage.^6^ Central fever has been defined as emergence of fever in patients with intracerebral hemorrhage without any focus of infection.^8^ Any disequilibrium in central thermomodulation relating to neurological damage may cause hyperthermia.^9^ In animal models, direct trauma applied to the preoptic nucleus of the hypothalamus has been observed to induce development of hyperthermia within two minutes.^10^ Central fever, which is a frequent complication seen in patients with intracerebral hemorrhage, has been seen in 72% of the patients diagnosed with subarachnoid hemorrhage, 37% of traumatic cerebral injuries and 32-37% of patients with a diagnosis of primary intracranial hemorrhage.^6^

If central fever seen in patients with intracerebral hemorrhage can be distinguished from manifestations of systemic inflammatory response syndrome (SIRS) due to infection, this will prevent use of inappropriate antibiotherapy and enable initiation of antibiotherapy that is effective for infection-related SIRS.^7^

Infectious and noninfectious causes of fever emerging in patients who have been hospitalized in an ICU need to be identified, and treatment should be rapidly instituted. Many studies have demonstrated that PCT levels do not change or only slightly increase in cases of noninfectious inflammation, surgical trauma, uncomplicated infection, autoimmune disease or neoplastic disease. Therefore, PCT can be used as a reliable biomarker for differentiating between bacterial and non-bacterial inflammatory processes.^11^^,^^12^^,^^13^^,^^14^

A significant correlation exists between increased PCT levels and greater severity of infection. Plasma PCT concentrations of between 0.5 and 2 ng/ml are deemed to be mildly elevated levels and are interpreted as local infection. PCT levels above 10 ng/ml are considered to be increased levels. PCT levels of up to 100 ng/ml are considered to be very high levels. Very high levels of PCT are seen in severe bacterial infections and in the hyperinflammatory phase of sepsis. In nonbacterial or nonparasitic diseases, PCT levels are generally below 2 ng/ml. In severe bacterial infections and sepsis, plasma PCT concentrations range between 2 ng/ml and 1,000 ng/ml.^15^

## OBJECTIVES

The aim of the present study was to investigate the relationship between PCT levels and both fever etiology and CRP levels among critically ill patients with intracerebral hemorrhage. Early determination of the etiology of their fever would enable rational use of antibiotics. Furthermore, through diagnosing infections at an early stage, morbidity and mortality would be prevented and there would also be economic gains.

## METHODS

### Study design and ethics

This cross-sectional study was conducted in accordance with the principles of the Helsinki Declaration and was approved by the local institutional review board (date: August 2, 2016; decision number: 155682).

### Setting and participants

Febrile patients admitted to Firat University Hospital ICU with a diagnosis of intracerebral hemorrhage (subarachnoid hemorrhage, traumatic subarachnoid hemorrhage, subdural hematoma or primary intracerebral hemorrhage) and normal procalcitonin levels (initially) between January 2015 and January 2017 were included in this study. Patients with symptoms of infection or histories of chronic rheumatic diseases (systemic lupus erythematosus, rheumatoid arthritis, familial Mediterranean fever, etc.) were excluded from the study.

### Outcome evaluations

Hemogram, ESR, biochemical tests, CRP levels and PCT levels were evaluated at baseline and then routinely every day, and the results were recorded. Procalcitonin values on the day of development of findings were recorded in relation to patients who developed signs and symptoms of infection.

Endotracheal aspirate cultures were obtained from patients connected to mechanical ventilation. Aspirator tip, urine and deep wound site cultures were obtained from patients who were not connected to mechanical ventilation. Cerebrospinal fluid cultures were obtained from operated patients with indwelling drains. The results from these cultures were all evaluated.

Infectious fever was defined as fever suggestively related to an infection, through evaluation of the patient’s clinical state and laboratory data by a specialist. The types of infections were classified according to the source of the infection, as blood stream, urinary, respiratory system or wound site infections.

Blood stream infection was defined as a situation in which a known pathogen isolated from one or more blood cultures was not associated with infection from another site or from growth of skin flora, in blood cultures obtained at two or more different time points. In catheterized patients, the following were defined as catheter-related blood stream infection: growth of the same microorganism both in semiquantitative peripheral blood cultures (> 15 colony-forming units/catheter segment) and in quantitative peripheral blood cultures (> 10^3^ colony-forming units/catheter segment); bacterial growth rate of > 5/1 in simultaneously obtained central venous catheter blood and peripheral blood cultures; or detection of bacterial growth at least two hours earlier in a blood culture obtained from a catheter, relative to a simultaneously obtained peripheral blood culture.

Urinary tract infection (UTI) was defined as one of the following: bacterial growth of > 10^5^ colony-forming units/ml or growth of at most two different bacteria in urine cultures; nitrite or leukocyte esterase positivity in complete urinalysis; or pyuria in a patient with one of the symptoms of fever, pollakiuria, dysuria or suprapubic tenderness.

The diagnosis of respiratory system infection was established based on a newly developed infiltration detected on chest X-ray, alteration to the patient’s respiratory function of the patient (increased ventilation support or oxygen requirement), increased purulence of aspirated secretion and positivity of sputum culture.

Presence of a wound site, purulent discharge, local pain and tenderness, local warmth, redness, swelling and bacterial growth in aseptically collected discharges or tissue cultures favored the presence of wound site infection. Patients in whom infection was detected in multiple foci were included in a multiple infection group.

Central fever in patients with a diagnosis of intracerebral hemorrhage was evaluated based on clinical, laboratory and culture results and was defined as absence of any infection.

The clinical progression of the patients, including their ICU stay, discharge and death, was also recorded on forms.

### Statistical analysis

The data were analyzed using the IBM Statistical Package for the Social Sciences v22 (SPSS, Inc., Chicago, IL, USA). The sample size was decided in accordance with a power analysis (significance level of P < 0.05; power analysis 80%). Descriptive statistics, including frequencies and percentages for categorical variables and the mean (± standard deviation) and median (with interquartile range, IQR) for continuous variables, were used to describe the baseline demographic data and clinical characteristics.

The variables were investigated using visual methods (histograms and probability plots) and analytical methods (Kolmogorov-Smirnov and Shapiro-Wilk tests) to determine whether or not they were normally distributed. The Mann-Whitney U test was applied to compare continuous variables. To determine the correlation between two continuous variables, Spearman’s rank correlation analysis was used for asymmetrical variables.

The data distribution was not normal, and for this reason, nonparametric analytical methods (Mann-Whitney and Spearman) were used. The cutoff values of PCT, CRP and ESR for predicting infectious fever were determined by means of a receiver operating characteristic (ROC) analysis. ROC curves were generated by plotting the relationship between true positivity (sensitivity) and false positivity (1-specificity) at various cutoff points of the tests. P-values < 0.05 were considered to be statistically significant in all analyses.

## RESULTS

A total of 98 patients were admitted to the ICU with a diagnosis of intracerebral hemorrhage. Among these patients, eight (8.16%) had abnormal procalcitonin levels, one (1.02%) had a history of rheumatic disease and 16 (16.32%) were afebrile on the day of admission. Therefore, all these patients were excluded from the study. Forty-five male patients (61.6%) and 28 female patients (38.4%) with a mean age of 55.59 ± 16.04 years (range, 22-87 years) were included in this study (total of 73 patients). They presented the following conditions: subarachnoid hemorrhage (n = 42; 57.5%), subdural hematoma (n = 15; 20.5%), traumatic subarachnoid hemorrhage (n = 12; 16.4%) and primary intracerebral hemorrhage (n = 4; 5.5%).

Among these 73 patients who were assessed, 39 (53.4%) presented infectious fever and 34 (46.6%) had central fever. The foci of infection were investigated. There were 22 cases of single-site infection, which most frequently consisted of respiratory tract infection (n = 17), followed by urinary tract infection (n = 3). The other 17 patients with infectious fever presented multiple infections.

The median PCT value among the 39 febrile patients with intracerebral hemorrhage who were diagnosed as presenting infectious fever was 4 ng/ml (IQR, 0.9-11). In the central fever group (n = 34), the median PCT was 0.1 ng/ml (0.1-0.4) (P < 0.001). PCT values were investigated according to the foci of infection, and the highest PCT values were found in patients with multiple infections: 5.0 ng/ml (2.7-27) (Table 1).

**Table 1.** Median procalcitonin (PCT) levels (ng/ml) of cases with infectious fever, according to focus of infection

Group	n (%)	PCT median (interquartile range)
Multiple infection sites	17 (43.6)	5.0 (2.7-27.0)
Single infection site	22 (56.4)	3.4 (0.8-6.8)
Pneumonia	17 (43.6)	4.3 (1.5-8.5)
Urinary tract infection	3 (7.6)	0.7 (0.5-0.8)
Wound site infection	1 (2.6)	1
Primary blood stream infection	1 (2.6)	0.8
Total	39 (100)	4 (0.9-11)

Multiple infections were investigated according to their foci of infection, as concomitant infections. These included secondary blood stream infections (total n = 15), pneumonia (n = 9; 52.4%), urinary system infections (n = 4; 23.53%) and wound site infections (n = 2; 11.76%). In one patient (5.88%), pneumonia and wound site infection were detected, and in another patient (5.88%), pneumonia and urinary tract infection.

The median PCT levels according to foci of multiple infections were 5.01 ng/ml (min-max, 0.1-200) in cases of blood stream infection plus pneumonia; 5.5 ng/ml (3.5-19) in cases of blood stream infection concomitant to urinary system infection; 5.01 ng/ml (5-12) in cases of blood stream infection plus wound site infection; 32.5 ng/ml in a patient with pneumonia plus urinary system infection; and 0.4 ng/ml in another patient with pneumonia plus wound site infection.

The median hospital stay among all the patients was 21 days (IQR, 13-47.5). For 36 patients with PCT > 0.5 ng/ml, it was 30 days (min-max, 5-210); while for those with PCT < 0.5 ng/ml, it was 20 days (min-max, 7-150). In parallel with longer hospital stays, increases in mean serum PCT levels were seen. However, no statistically significant differences were detected (P = 0.051).

The median PCT level of patients who died (n = 28; 38.36%) was 5 ng/ml (IQR, 0.8-18.5). This was significantly higher than that of discharged patients (n = 45; 61.64%), for whom the PCT level was 0.2 ng/ml (IQR, 0.1-0.6) (P < 0.001).

The median CRP level among the patients with infectious fever was 167 mg/l (IQR, 138-201); while the median CRP level of the cases with central fever was 72.5 mg/l (IQR, 16.25-137.7).

**Figure 1.**
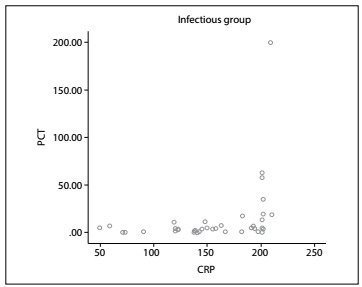
Correlation between procalcitonin (PCT) and C-reactive protein (CRP) levels in cases with infectious fever.

A statistically significant positive correlation was detected between PCT and CRP levels in patients with infectious fever (rho: 0.461; P = 0.003) (Figure 1). However, in cases with central fever, no significant correlation was detected between PCT and CRP levels (rho: 0.239; P = 0.173). In both groups, there were no statistically significant correlations between PCT levels, ESR, blood leukocyte levels and polymorphonuclear leukocyte (PMNL) percentages.

The median white blood cell count, neutrophil count, erythrocyte sedimentation rate, CRP level and PCT level of the cases are presented in Table 2. A statistically significant difference was detected between the infectious fever and central fever groups regarding PCT level, ESR and CRP level.

**Table 2.** Laboratory parameter results from the infectious fever and central fever groups, expressed as median (interquartile range)

	Infectious fever (n = 39)	Central fever (n = 34)	P
Blood leukocyte count (10^3^/µl)	11200 (7550-13500)	10625 (6545-12125)	0.278
Blood PMNL percentage (10^3^/µl)	80 (76-87)	79 (66-84)	0.121
Erythrocyte sedimentation rate (mm/h)	90 (65-105)	55 (30-77)	< 0.001
C- reactive protein (mg/l)	167 (138-201)	72 (16-137)	< 0.001
Procalcitonin (ng/ml)	4 (0.9-11)	0.1 (0.1-0.4)	< 0.001

PMNL = polymorphonuclear leukocyte.

**Figure 2.**
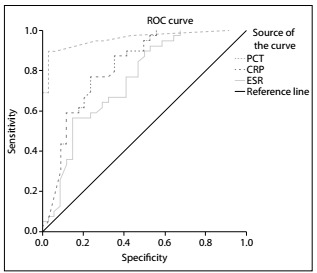
Receiver operating characteristic (ROC) curves for procalcitonin (PCT), C-reactive protein and erythrocyte sedimentation rate (ESR), for predicting diagnoses of infectious fever in cases of intracerebral hemorrhage.

To evaluate the sensitivity and specificity of these three biomarkers, ROC curves were calculated. The ROC analysis showed that PCT was a good marker for distinguishing infectious fever patients, with an area under the curve (AUC) of 0.958 (95% confidence interval: 0.912-1.000; P < 0.001). CRP was found to have an AUC of 0.816 (95% confidence interval: 0.714-0.917; P < 0.001). The ESR was found to have an AUC of 0.748 (95% confidence interval: 0.633-0.863; P < 0.001). The sensitivity and specificity of PCT, CRP and ESR for diagnosing infectious fever in cases of intracerebral hemorrhage are presented in Table 3. ROC curves comparing PCT, CRP and ESR with regard to predicting the diagnosis of infectious fever in cases of intracerebral hemorrhage are shown in Figure 2.

**Table 3.** Sensitivity and specificity of procalcitonin (PCT), C-reactive protein (CRP) and erythrocyte sedimentation rate (ESR) for diagnosing infectious fever in cases of intracerebral hemorrhage

	PCT	CRP	ESR
Cutoff	0.35 (ng/ml)	119.5 (mg/l)	56.5 (mm/h)
Sensitivity	94.87	84.62	87.18
Specificity	73.58	64.71	50.0
Positive predictive value	80.43	73.33	66.67
Negative predictive value	92.59	78.57	77.27
Area under the curve	0.958	0.816	0.748
P-values	< 0.001	< 0.001	< 0.001

## DISCUSSION

Recently, the information available about use of PCT as a biomarker in ICUs has been increasing. In the literature, most PCT-related studies have focused on PCT levels relating to the diagnosis of sepsis. The objectives of investigating PCT levels have been to detect possible bacterial infection in order to reveal the severity of SIRS, discern the progression of infection towards sepsis, observe the response of sepsis to treatment and predict the prognosis.^16^

It has been demonstrated that PCT levels enable reliable differentiation of sepsis from SIRS.^17^ In both human and animal models, increased levels of PCT production have been demonstrated in situations of bacterial infection.^18^ PCT levels markedly increase when bacterial infection is present; while in situations of localized or viral infections, PCT levels remain normal or increase slightly. Festic et al. conducted a study on patients who were followed up in ICUs with a diagnosis of subarachnoid hemorrhage. They evaluated PCT levels at all foci of infection and noted that PCT had high specificity but low sensitivity for predicting infection. However, when UTIs were considered to be minor infections and were excluded from the analysis, it was then observed that PCT levels presented increased specificity and sensitivity in relation to infection.^19^

Halvarson et al. conducted a study on 73 patients with diagnoses of spontaneous subarachnoid hemorrhage, subdural hematoma, traumatic subarachnoid hemorrhage or primary intracerebral hemorrhage who had been hospitalized and were being followed up in the ICU of a neurology department. These authors investigated the specificity of PCT levels for differentiating between infectious and central fevers. The patients were classified according to their foci of infection, as patients with pneumonia, urinary tract infection, infection of the bloodstream, encephalitis, sinusitis, enterocolitis or multiple infections.^20^ PCT levels were compared between patients with infectious and central fevers, and increased PCT levels in cases of intracerebral hemorrhage were not found to be specific for the diagnosis of infectious fever. In their study, localized infections, such as sinusitis and enterocolitis, were frequently encountered; however, in our study, higher incidence of secondary bloodstream infections was encountered, which may have been the reason for the relatively higher PCT levels.

A relatively small-scale study in which PCT was investigated as a biomarker of infection was unable to provide adequate and significant information on this issue.^21^ In another study, PCT levels were compared between patient groups with sterile meningeal inflammation in Neuro-Behcet’s disease and with bacterial meningitis, and no statistically significant difference was found.^22^ The differences that have arisen between various studies may have been due to the diverse effects of different types of infection on the production of PCT. Larger-scale studies need to be conducted on this issue.

Several investigations have been performed concerning the use of CRP and PCT levels for diagnosing and following up cases of infection in patients. In most studies, PCT has been reported to be superior to CRP for differentiating between sepsis and SIRS. However, some other studies have not indicated any superiority of PCT.^23^^,^^24^^,^^25^^,^^26^^,^^27^ PCT has been found to be 7% more sensitive and 23% more specific than CRP for establishing the diagnosis of bacterial infection.^28^^,^^29^ In another study, it was reported that both PCT and CRP could be used in making the diagnosis of infection, but that PCT was superior for determining the prognosis.^30^

Ugarte et al. followed up 190 adult ICU patients and diagnosed the presence of hospital-acquired infection in 111 patients. They compared the patients with and without infection and observed that there were statistically significantly higher PCT and CRP levels in patients with infection.^31^ In their study, the best cutoff values for PCT and CRP were 0.6 ng/ml and 7.9 mg/dl, respectively. According to their study, PCT is not a better marker of infection than CRP, among critically ill patients.

In our patients, a statistically significant positive correlation was detected between the PCT and CRP levels in cases with infectious fever. However, this significant correlation was not detected in the central fever group (rho: 0.239; P = 0.173). CRP is classified as an acute-phase reactant, which means that its levels will rise in response to inflammation. A variety of conditions could commonly cause increases in the levels of CRP and other inflammatory markers.

The limitations of the present study included its characteristics of being a single-site study with only a small number of patients. Multi-site studies would enable acquisition of more accurate information.

## CONCLUSIONS

Differentiation between infectious and noninfectious etiologies of fever that emerges in patients who have been diagnosed with intracerebral hemorrhage, along with early institution of treatment, makes it possible to institute appropriate antibiotic therapy in patients with infection and prevent unnecessary use of antibiotics in patients with central fever. This adds importance to the guiding role of serum PCT levels in making the diagnosis and prognosis of infection. In this population, serum PCT levels can help physicians to correctly diagnose and use antibiotics. Our study demonstrated that PCT levels can be used to differentiate between infectious and central fevers in patients with intracerebral hemorrhage, with high specificity and predictive values for infection. However, studies with larger numbers of patients need to be conducted in relation to this issue.
