# ERRATUM

**DOI:** 10.1590/1516-3180.2019.ast.0458051119erratum

**Published:** 2020-01-13

At the request of the authors, we report that in the paper published in the Sao Paulo Medical Journal, volume 137, issue number 4, DOI: 10.1590/1516-3180.2018.0458220719, pages 349-55 (in the title):

**Where it read:**

“Procalcitonin levels among patients with fever secondary to severe intracerebral infection. A cross-sectional study”

**It should read:**

“Procalcitonin levels among patients with fever secondary to intracerebral hemorrhage and severe infection. A cross-sectional study”

